# FKBP51 Immunohistochemical Expression: A New Prognostic Biomarker for OSCC?

**DOI:** 10.3390/ijms18020443

**Published:** 2017-02-18

**Authors:** Daniela Russo, Francesco Merolla, Massimo Mascolo, Gennaro Ilardi, Simona Romano, Silvia Varricchio, Virginia Napolitano, Angela Celetti, Loredana Postiglione, Pier Paolo Di Lorenzo, Luigi Califano, Giovanni Orabona Dell’Aversana, Fabio Astarita, Maria Fiammetta Romano, Stefania Staibano

**Affiliations:** 1 Department of Advanced Biomedical Sciences, University “Federico II”, 80131 Naples, Italy; danielarusso83@yahoo.it (D.R.); mmascol@gmail.com (M.M.); gennaro.ilardi@gmail.com (G.I.); silvia.varricchio@gmail.com (S.V.); virnap87@hotmail.it (V.N.); pierpaolo.dilorenzo@unina.it (P.P.D.L.); 2 Department of Medicine and Health Sciences “V. Tiberio”, University of Molise, 86100 Campobasso, Italy; francesco.merolla@unimol.it; 3 Department of Molecular Medicine and Medical Biotechnology, University “Federico II”, 80131 Naples, Italy; romanos@dbbm.unina.it (S.R.); mariafiammetta.romano@unina.it (M.F.R.); 4 Institute for the Experimental Endocrinology and Oncology, Research National Council, 80131 Naples, Italy; celetti@unina.it; 5 Department of Translational Medical Sciences and Center for Basic and Clinical Immunology Research, University “Federico II”, 80131 Naples, Italy; loredana.postiglione@unina.it; 6 Department of Maxillofacial Surgery, University “Federico II”, 80131 Naples, Italy; califano@unina.it (L.C.); giovanni.dellaversanaorabona@unina.it (G.O.D.); dottastarita@gmail.com (F.A.)

**Keywords:** FKBP51, oral cancer, prognosis, immunotherapy, Bayes theorem, squamous cell carcinoma

## Abstract

Up-to-date, several molecular markers of prognosis have been studied in Oral Squamous Cell Carcinoma (OSCC), but none entered in the clinical setting. Therapy of OSCC tumors mainly relies on surgery, radiotherapy and partially on chemotherapy; there is an urgent need for biomarkers able to better stratify OSCC patients’ risk to address targeted therapeutic strategies. The role of immune response in the pathogenesis and biological behavior of OSCC has been investigated by several authors, and promising results have been obtained with immune checkpoint inhibitors. We already investigated the role of the immune modulator FK506-binding protein 51 (FKBP51), a FK506-binding immunophilin, in cutaneous melanoma biology, and its expression in several human solid tumors. In the present study, we aimed to assess the value of FKBP51 expression in OSCC tumor cells as a marker of outcome. We collected clinical data from 72 patients who underwent surgery for Squamous Cell Carcinoma (SCC) of the tongue, floor, lips and palate. FKBP51 expression was assessed by immunohistochemistry on paraffin-embedded tumor tissues. In addition, we evaluated the human papillomavirus (HPV) status of primary tumors by immunohistochemistry, viral subtyping and In Situ Hybridization (ISH) assay. We found that high FKBP51-expressing tumors characterized the OSCCs with the worst prognosis: the high immunohistochemical expression of FKBP51 associated with death occurring within five years from the diagnosis with a sensitivity of 88.46% and a specificity of 91.67%. The estimated positive predictive value of the test was 88.45% and negative predictive value 91.67%. We tested FKBP51 mRNA presence, by RT-PCR assay, in a selected series of OSCC tumors, and we found that mRNA correlated well to the protein expression and to the clinical outcome. Applying the Bayes formula, we estimated an 88% probability of dying within five years from the diagnosis of OSCC patients with a high FKBP51 immunohistochemical (IHC) test result (>51% of FKBP51 positive tumor cells). On the basis of our analysis, we propose tumor tissue expression of FKBP51 protein as a reliable prognostic marker for OSCC tumors.

## 1. Introduction

Head and neck cancers (HNCs) include a broad range of malignancies that mainly originate from the lining epithelium of the nasal cavity, paranasal sinuses, nasopharynx, oral cavity, oropharynx and larynx and mostly consist of squamous cell carcinomas (head and neck squamous cell carcinoma (HNSCC)) [1]. Squamous cell carcinomas of the oral cavity (OSCC) represent the most frequent forms of HNSCCs. About 50,000 new cases of oral cancers and 10,000 deaths have been estimated in 2016 in the USA; the global incidence is about 300,000 new cases per year with an annual mortality rate of about 145,000, Melanesia, South-Central Asia and Central-Eastern Europe accounting for the highest rates [2,3]. According to the national cancer registry data, in Italy, there will be about 10,000 new head and neck cancer cases in 2016; the risk of developing a tumor is much higher in males than females; and the prevalence of the disease is higher than 100,000 cases per year [4]. In the last 30 years, there has been a noticeable change in the epidemiology of HNCs, this leading some authors to refer to it as “The New Face of HNCs” [5,6]. OSCC is classically associated with a history of alcohol and tobacco (either smoked or chewed) abuse, but its etiological correlation with human papilloma virus (HPV) infection is well established, as well. In fact, although the exposure to classical causative agents such as alcohol and tobacco remains the largest risk factor for squamous cancers of the aerodigestive tract, a rapid expansion of OSCC related to oncogenic HPV infection is still occurring, whose spreading in some regions has been referred to as epidemic [3]. In 2020 in the USA, it is expected that the annual incidence of HNCs will surpass that of the cervical cancer related to HPV infection.

Currently, prognostic and predictive factors able to give indications about the biological aggressiveness of the OSCC are very few and essentially related to the tumor size and therapy response. Persistent HPV infection is a well-assessed causative event in oropharyngeal squamous cell carcinogenesis [7,8], and tumor HPV status is a strong and independent prognostic factor for survival of oropharyngeal Squamous Cell Carcinoma (SCC) patients [9], while the role of HPV infection in non-oropharyngeal SCC is still debated [10,11]. To date, beyond HPV, there are no reliable prognostic or predictive biomarkers, especially for non-oropharyngeal SCC [12,13].

Our group, in the last decade, has identified a relevant role in chemo- [14] and radio-therapy [15] resistance for the large immunophilin FK506 binding protein 51 (FKBP51). In addition, particularly for melanoma, we have for the first time disclosed an association between FKBP51 expression, as determined by immunohistochemistry and tumor aggressiveness [15].

FKBP51 (*FKBP5* gene) is a large molecular weight component of the family of FK506 binding proteins (FKBPs), classically known as the intracellular receptors for immunosuppressants FK506 and rapamycin [16,17]. FKBPs are multifunctional proteins that modulate several signal transduction pathways [16,17] and often exploited by cancer cells, in an opportunistic manner, to support its needs for growth and survival [18].

To sort out a new biomarker able to predict the OSCC biological behavior, we focused our attention on the *FKBP5* gene product. To this aim, we studied FKBP51 protein expression in a series of OSCC by immunohistochemistry. In addition, we related our data to the HPV status of primary tumors, by immunoexpression of p16^INK4a^ protein. Finally, given an IHC test resulting in a high FKBP51 phenotype, we quantified the risk of a poor outcome per FKBP51 protein expression calculating the probability of the occurrence of patient death. Our study supports the conclusion that a positive correlation subsists between FKBP51 expression and the poor outcome of OSCC.

## 2. Results

### 2.1. Study Population

The clinicopathological characteristics of the study population are summarized in Table 1. Out of 72 cases, 40 male and 32 female, the age at diagnosis ranged between 29 and 89 years (mean age 63.8, median 64).

**Figure 1:**
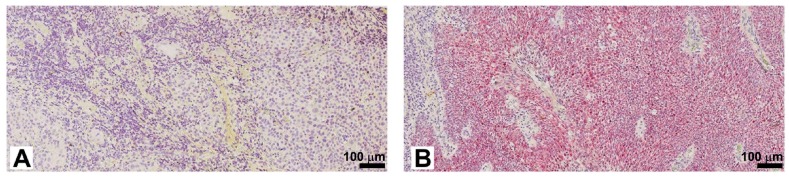
Two representative images of ISH analysis (RNAScope) showing non-actively replicating HPV virus (**A**); and an active replicating HPV virus (**B**). Scale bar: 100 μm.

The histotype was SCC for all tumors under investigation; the most affected site was tongue; 26 out of 72 were oropharyngeal squamous cell carcinomas, the remaining 46 originated in other sites of the oral cavity. Six out of 26 oropharyngeal tumors were HPV positive, while all of the non-oropharyngeal ones were HPV negative. The p16^INK4a^-positive tumors were all positive for HPV16 genotype. HPV positivity was confirmed by In Situ Hybridization (ISH) analysis (RNAScope), demonstrating that virus was actively replicating [19] (Figure 1).

The TNM tumor pathologic stage of all cases was determined based on the clinic-pathological information, according to the American Joint Committee on Cancer (AJCC) 7th edition [20]. Based on the histopathological examination, three tumors were classified as well differentiated; 23 were moderate; and 40 were poorly differentiated.

During the observation time, seven patients were lost at the follow-up, and so, their data were censored from the statistical analysis. During the follow-up period (mean of 40.9 months), out of 66, 33 patients remained tumor-free and 32 patients died from disease; seven patients showed metastasis and 17 patients recurrence.

### 2.2. Immunohistochemical Staining and Statistical Analysis

**Figure 2:**
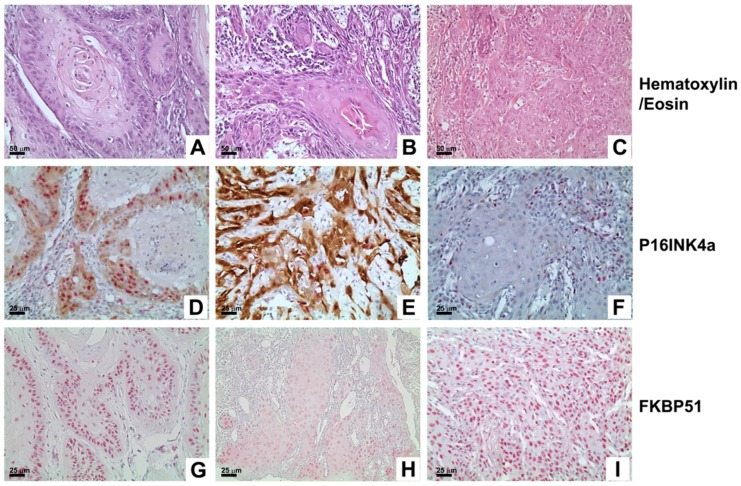
Oral Squamous Cell Carcinoma (OSCC), three representative cases: (**A**–**C**) Hematoxylin/eosin stain (200×; scale bar: 50 µm); (**D**–**F**) P16^INK4a^ stain (400×; scale bar: 25 µm); (**G**–**I**) FKBP51 stain (400×; scale bar: 25 µm), respectively low expression (30%), medium (50%) and high expression (95%).

FKBP51 expression was evaluated as the percentage of positive tumor cells: for each sample, the percentage of FKBP51 positive cells was counted on 10 high power fields (HPF) (Figure 2). FKBP51 positivity ranged between 0% and 100% of tumor cells, mean value 48.2% (95% Confidence Interval CI for the mean 41.4%–54.9%), median value 51% (95% CI for the median 33.9%–70%).

**Figure 3:**
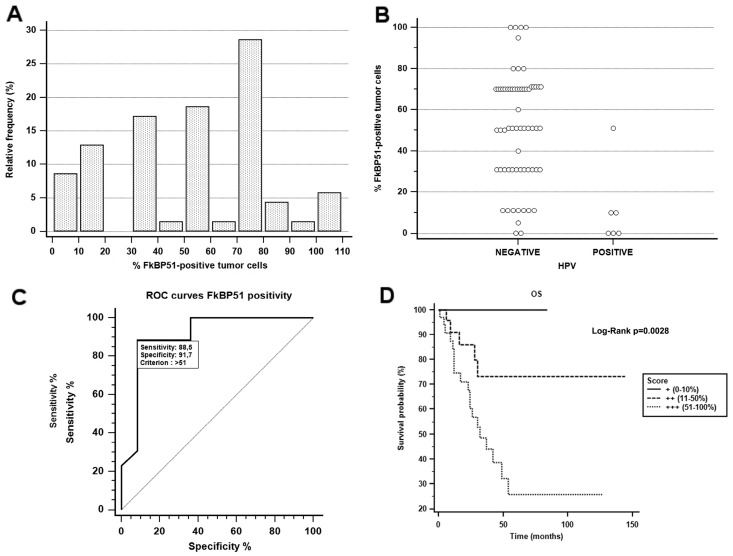
(**A**) Relative frequency of FKBP51 % of positive tumor cells in the case population studied; (**B**) p16^INK4a^ staining plotted against FKBP51 IHC results; (**C**) FKBP51 IHC expression ROC curves; (**D**) Kaplan–Meier curves. Patients were grouped into three risk categories according to FKBP51 expression (+: 0%–10%; ++: 11%–50%; +++: 51%–100%).

The relative frequency distribution of values is shown in Figure 3A. The quantitative assessment of FKBP51 IHC positivity in the study population cases, expressed as the percentage of FKBP51-positive tumor cells, was used to determine the sensitivity and specificity of the test relative to death within the first five years from diagnosis. To this aim, Receiver Operating Characteristic (ROC) curves were drawn, and the analysis gave the following results: the area under the ROC curves was 0.907 with a 95% CI ranging between 0.806 and 0.966 (*p*-value < 0.0001). At the criterion value >51 (%FKBP positive tumor cells) the sensitivity and specificity of the test were 88.46 (95% CI 69.8–97.6) and 91.67 (95% CI 77.5–98.2), respectively (Figure 3C). The prevalence of the event being 41.9%, we estimated the positive predictive value at 88.4% (95% CI 69.8–97.6) and the negative predictive value at 91.7% (95% CI 77.6–98.2) (Table 2).

In order to test the accuracy of the FKBP51 IHC test, we applied a Markov chain Monte Carlo (MCMC) model that allowed us to simulate the sensitivity of the test over thousands of iterations [21].

**Figure 4:**
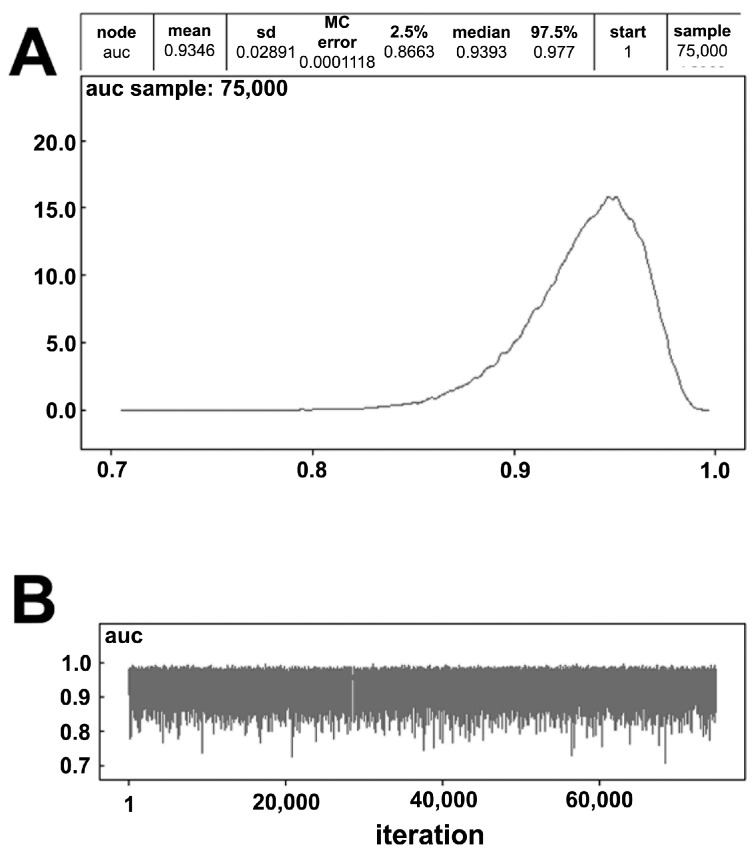
(**A**,**B**) Graphical representation of the Markov chain Monte Carlo (MCMC) model sensitivity simulation over 75,000 iterations.

The results of our MCMC analysis is shown in Figure 4: the good accuracy of the test was confirmed ad proven to be even better in MCMC simulation (Figure 4).

By Kaplan–Meier curves analysis, survival estimates have shown that OSCCs overexpressing FKBP51 (cut-off >51%) are characterized by a worse prognosis, in terms of overall survival. The risk stratification allowed us to group the study population into three categories based on the FKBP51 positive tumor cells (+: 0%–10%; ++: 11%–50%; +++: 51%–100%). Comparison of survival curves proved to be statistically significant for the log-rank test (*p* = 0.0041) (Figure 3D).

Mean FKBP51 expression in the six HPV-positive tumors, all of them showing good outcome, were 11.8%, against an average positivity of 59.6% in the remaining 20 HPV negative oropharyngeal tumors (*p* < 0.05 ANOVA test); average FKBP51 positivity in the 46 non-oropharyngeal tumors was 48% (Table 3 and Figure 3B).

### 2.3. Bayesian Statistic

In order to quantify the probability of death within the first five years from the diagnosis given the IHC test for FKBP51-positive tumor cells (>51% of FKBP51 positive tumor cells), we applied the Bayes formula:

$$p\left(A|B\right)=\frac{p\left(B|A\right)p\left(A\right)}{p\left(B\right)}$$

*A*: the adverse event (i.e., death within five years from diagnosis), this is the prior distribution of our analysis and corresponds to the prevalence of the event that we estimated at 41% based on the literature data. This value is consistent with the rate of deadly outcomes we could observe also in our study. *B*|*A* (*B* given *A*): the rate of positive tests when the event is present is basically the sensitivity of the test, and in our case is 88.46%. *B*: the rate of “positive” FKBP51 IHC tests (>51% of positive tumor cells).

We derived the *p*(*B*) as follows:

p(B)=p(B|A)×p(A)+[p(B|(1−A)]×p(1−A)

The probability of getting a positive test result is *p*(*B*) = 0.363. *A*|*B* (*A* given *B*): the rate of the event occurring when the test result is positive. Applying the Bayes formula, this parameter was 88.10%, that is the probability an OSCC patient will die within five years from diagnosis having a positive FKBP51 IHC test result.

### 2.4. FKBP51 mRNA Expression

**Figure 5:**
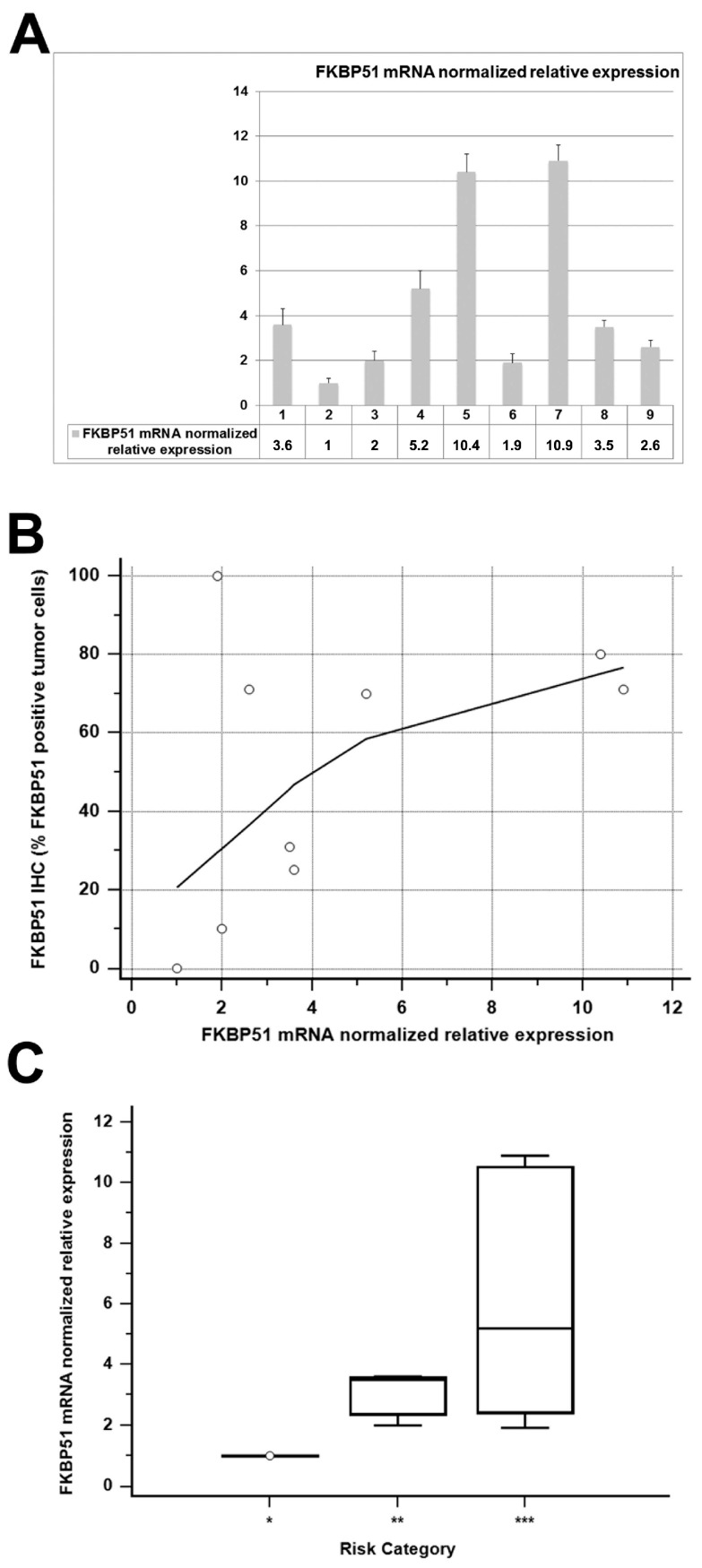
(**A**) FKBP51 mRNA normalized relative expression in nine representative cases; (**B**) scatter plot showing the relationship between FKBP51 mRNA normalized relative expression and FKBP51 IHC results (expressed as % of positive tumor cells); (**C**) box plot sowing the FKBP51 mRNA normalized relative expression distribution over the three patients’ risk categories (*, **, *** as defined by survival curves in Figure 3D).

FKBP51 expression in tumor samples was assessed also by real*-*time PCR to establish whether FKBP51 expression was regulated at the transcriptional level in OSCC. Nine representative OSCC tumor samples, tested both for IHC and real-time PCR (Figure 5A), showed a strong concordance of FKBP51 protein and mRNA presence in OSCC tumors (Figure 5B).

Moreover, a strong tendency of correlation was observed between FKBP51 normalized relative mRNA expression and patients’ risk category (Figure 5C).

## 3. Discussion

Head and neck cancers are mostly squamous cell carcinomas of the oral cavity; although the most important risk factor is tobacco (both smoked and chewed) and alcohol consumption, HPV infection has emerged as a causative event in a growing number of cases worldwide [22].

Most authors described two distinct diseases, different in pathogenetic events and clinical outcome: an HPV-related oropharyngeal squamous cell carcinoma and a tobacco/alcohol-related oral squamous cell carcinoma (especially of non-oropharyngeal origin). This dichotomy is debated in light of the evidence of a cross-talk between the two mechanism, and the immune response elicited both by HPV infection and by smoking and alcohol could provide a common field to explain the mutual influence between the two etiopathogenetic events.

Treatment of OSCC patients often involves many specialists due to the anatomic complexity of the region and the therapeutic approaches in the current practice for advanced diseases (surgery, radiotherapy, chemotherapy, plastic reconstruction for aerodigestive function preservation) [1].

During the last few decades, we have witnessed many advances in intensive treatments of OSCC; induction chemotherapy, hyperfractionated radiotherapy, surgery and EGFR inhibitors are currently applied to OSCC patients, unfortunately carrying severe acute and chronic side effects, heavily impacting on patients’ quality of life. Too many times, we have seen patients over treated due to the lack of good prognostic markers; intensive treatment should be used exclusively on patients positive for the expression of markers associated with poorer outcome [23].

In clinical-pathological practice, it is therefore difficult to segregate patients into the correct classes of risk; in an era of evolving precision medicine, the emergence of HPV infection as a cause of OSCC may represent a predictive biomarker and therefore is considered of prognostic value in oropharyngeal SCC [9]; immunohistochemical detection of surrogate biomarkers (e.g., P16^INK4a^ protein) has been advocated as the best test to use for risk stratification in OSCC [24,25,26,27,28], although the point is still debated [29,30,31].

However, at present, no reliable biomarkers exist to predict the outcome of HPV unrelated OSCC, although, up-to-date, several molecular markers of prognosis have been studied in OSCC, but none entered in the clinical settings [32,33].

The expression of the *FKBP51* gene was reported to be altered in a relevant number of malignancies [34]. The gene has been found either hypo-expressed or hyper-expressed in several cancers, with contrasting results concerning its biological significance. As an example, high expression levels of FKBP51 have been related with either the suppression or promotion of tumor growth, depending on the specific tumor type and its relative microenvironment. The low expression level in pancreatic cancer cell lines and tumor tissue has been recently correlated with the hypothesized loss-of-function of FKBP51 as a tumor suppressor in the context of the AKT signaling pathway. In colon cancer, IHC was of great importance to confirm the molecular findings, evidencing the presence of a clear cytoplasmatic signal, with any significant difference between normal and neoplastic tissues. In our hands, we showed a potential oncogenic effect of FKBP51 overexpression in human cutaneous melanoma (CMM). In >70% of cases, the neoplastic melanocytes of the non-invasive (radial) growth phase of CMM showed a low immunopositivity, whereas a stronger signal was found in tumor cells of the invasive (vertical) growth phase of all CMM. The highest immunoreactivity for FKBP51 was found in all of the metastatic melanoma cases. This prompted us to postulate a definite role for FKBP51 as a novel marker [35] of melanocyte malignancy. We moreover examined a series of 30 prostate cancers by immunohistochemistry and found a more intense signal FKBP51 in tumors with a high Gleason grade in comparison to the well-differentiated ones [34,36].

In the present paper, we found a significant correlation between FKBP51 expression and outcome of the oral squamous cell carcinomas.

The ROC curve analysis gave us a significant value of *p* (<0.0001) in the evaluation of the AUC (equal to 0.907 for our data). ROC curves allowed us to fix the critical value of positivity for FKBP51 by 51% in relation to which we obtained the sensitivity and specificity of the test in identifying patients at risk of death within five years of diagnosis. The positive predictive value and negative predictive value of the test were equal to 88.45% and 91.68%, respectively.

The analysis of survival curves allowed us to divide the sample into three categories of risk on the basis of outcome. The survival curve with the best prognosis includes individuals whose tumors show a level of positivity for FKBP51 rated from 0%–10%; the intermediate outcome is attributed to levels of positivity for the marker from 11%–50%; while a higher value of 51% positive tumor cells is sufficient to identify the group of patients with the worst prognosis at follow-up. The difference between the three curves was extremely significant; in fact, the value of *p* assigned by the log-rank test is equal to 0.0041.

Based on the determination of the values of *p*, the first conclusions we draw from the statistical analysis of the data are: (i) FKBP51 IHC expression inversely correlates with tumor HPV status both in the overall study population (*p* = 0.0026) and particularly in the oropharyngeal samples (*p* < 0.0001); (ii) patients with OSCC can be divided into three categories of risk depending on the positivity to the marker FKBP51. The value of 51% positive tumor cells, as well as being the critical value resulting from the analysis of ROC curves, is also the discriminant of the definition of the category at greater risk of death within the first five years after diagnosis.

Despite that the results obtained so far are comforting, we wanted to add to the analysis described above an estimate of the conditional probability of experiencing an adverse event, computing it according to Bayes’ theorem.

On the basis of our results, the probability that a patient operated for OSCC dies in the first five years from diagnosis, given a FKBP51 tumor positivity >51%, is 88.10%, so about 49 percentage points higher than the expected rate, that is about 39% according to the epidemiology.

These data allow us to look at the clinical use of our marker with great confidence and, at the same time, to have a reference value that is very easy to understand even for non-professionals, such as, in our experience, that they are the same patients in almost all cases.

The critical analysis of our judgment based on the calculation of the conditional probability using a Bayesian inference technique requires us to highlight some important aspects. What we present here is the first work that describes the association between tissue expression of FKBP51 and the outcome of OSCC. The literature described the potential role of the oncogenic protein; we ourselves have contributed to the functional characterization and expression of FKBP51 in melanoma, but to date, there are no reports in the literature on the relationship between FKBP51 and OSCC. For this reason, we do not have a precedent to look at to use in our analysis, and it is for this reason that the sensitivity of the test values and the frequency of positivity of the same, included in the Bayes formula, are obtained by the present study. The situation is different with regard to the rate of adverse events associated with the disease; this figure is present in the literature and is estimated at around 39%. Of the same order of magnitude is the rate of deceased presence in our series, which is therefore in line with the values reported in the literature. We firmly believe that the statistical analysis of data should be conducted with more than one approach in order to return a picture of the results as complete as possible.

Our finding that FKBP51 is a marker for oral cancer aggressiveness can open the door to new treatment options of this neoplasia. Indeed, previous studies have demonstrated the role of FKBP51 in cancer chemo- and radio-resistance [37,38]. Recently, FKBP51-selective tool compounds, SAFit1 and SAFit2 [39], proved to effectively inhibit several FKBP51 functions, either neuropsychiatric, through stress hormone regulation [40] or pro-tumoral ones, through NF-κB signaling suppression [40]. Refinements in the design of these ligands are continuously in evolution for the optimization of drug structures to increase bioavailability without affecting selectivity [41]. Future studies will address the efficacy of these compounds in oral cancer in order to verify if, in combined therapy, they can allow lowering the doses of traditional therapeutic agents, thus reducing side effects, while improving treatment outcomes.

## 4. Materials and Methods

### 4.1. Patients and Tissue Samples

Formalin-fixed, paraffin-embedded tissue blocks of 72 primary OSCCs, diagnosed and excised with healthy surgical margins from January 2000–January 2016, were retrieved from the archives of the Pathology Section of the Department of Advanced Biomedical Sciences, “Federico II” University of Naples. The clinical data and pathological features of the tumors are reported in Table 1. No patient experienced radiotherapy before surgery. The study design and procedures involving tissue sample collection and handling were performed according to the Declaration of Helsinki, in agreement with the current Italian law, and to the Institutional Ethical Committee guidelines.

### 4.2. Immunohistochemistry

For each case, we selected a block of tissue fixed in formalin and embedded in paraffin representative of the tumor and used it to obtain serial sections. One section was stained with hematoxylin/eosin to confirm the initial diagnosis; the others were used for immunohistochemical investigation.

Immunohistochemical analysis was performed on 4 μm-thick serial sections, mounted on poly-l-lysine-coated glass slides. The sections were deparaffinized and subjected to antigen retrieval by microwave oven treatment (3 min × 3 times, in EDTA buffer, pH 8.0); the backdrop (for blocking non-specific background staining) was removed using the universal blocking serum (Dako Diagnostics, Glostrup, Denmark) for 15 min at room temperature. Endogenous alkaline phosphatase activity was quenched adding Levamisole to buffer AP (substrate buffer); the slides were rinsed with TRIS + Tween20 pH 7.4 buffer and incubated overnight at 4 °C in a humidified chamber with the primary antibody anti-FKBP51 (sc-13983, Santa Cruz Biotechnology, Santa Cruz, CA, USA) diluted 1:200. Then, a biotinylated secondary antibody and streptavidin conjugated with alkaline phosphatase were used. The reaction was highlighted with chromogen fast red, which showed the presence of the antigen that we sought in red (Dako REAL Detection System, Alkaline Phosphatase/RED, Rabbit/Mouse, Glostrup, Denmark). Again, after a weak nuclear counterstain with hematoxylin, the sections were then mounted with a synthetic medium (Entellan, Merck, Darmstadt, Germany).

Histological figures has been acquired with a Leica DFC290 microscope (Leica Biosystems Nussloch GmbH, Heidelberger, Germany) at the highest resolution and saved in TIFF. Then, images have been assembled with Adobe Photoshop CS6 software (Adobe Systems Incorporated, San Jose, CA, USA).

### 4.3. HPV Testing and Genotyping

Immunohistochemical screening/staining for HPV status was performed using the p16^INK4a^-CINtec Histology kit (E6H4, Roche, Basel, Switzerland). Positive cases of immunostaining for p16^INK4a^ were genotyped for HPV. HPV genotyping was performed using the INNO-LiPA HPV Genotyping v2 Extra assay (Fujirebio, Inc., Malvern, PA, USA), according to the manufacturer’s protocols.

### 4.4. Real-Time PCR

Total RNA was isolated from Formalin Fixed Paraffin Embedded (FFPE) tissue samples using TRIzol (Invitrogen, Carlsbad, CA, USA) according to the manufacturer’s instructions. One microgram of each RNA was used for cDNA synthesis with Moloney Murine Leukemia Virus Reverse Transcriptase (M-MLV RT, Invitrogen, Carlsbad, CA, USA). Gene expression was quantified by real-time PCR using iQ™SYBR^®^Green Supermix (Bio-Rad, Hercules, CA, USA) and specific real-time validated QuantiTect primers for FKBP51 (QT00056714: NM_001145775 800 and 900 bp; NM_001145776 650 and 750 bp; NM_001145777 650 and 750 bp; NM_004117 600 and 700 bp). Relative quantitation of the transcript was performed using co-amplified ribosomal 18S as an internal control for normalization. The ribosomal 18S primers were forward 5′-CGATGCGGCGGCGTTATTC-3′ and 18S reverse 5′-TCTGTCAATCCTGTCCGTGTCC-3′.

### 4.5. Statistical Analysis

The expression of FKBP protein was expressed as % of FKBP51 positive tumor cells. The statistical significance of differences of FKBP51 expressions between groups was determined by one-way analysis of variance (ANOVA).

ROC curves were used to assess the criterion value, sensitivity and specificity, positive predictive value and negative predictive value of the test.

Patients were stratified according to the risk of death at the follow-up by Kaplan–Meier survival curves analysis. For comparison of the event-free survival time (events: death) between three categories of individuals, the log-rank Mantel–Haenszel test was applied. A two-tailed test of significance with a *p*-value < 0.05 was considered statistically significant. Multivariate analyses were performed to correlate different markers.

The statistical analysis was performed using the r statistical package v. 2.10.1 (R Foundation for Statistical Computing, Vienna, Austria). Evaluation of the intraobserver and interobserver agreement for the tested proteins on whole sections was performed by use of Cohen’s weighted kappa statistic.

### 4.6. Bayesian Statistic

The conditional probability of death given a positive result of the IHC assessment of FKBP51 expression (>51% of FKBP51 positive tumor cells) was calculated with Bayes formula:

$$p\left(A|B\right)=\frac{p\left(B|A\right)p\left(A\right)}{p\left(B\right)}$$

*A*: the adverse event (i.e., death within 5 years from the diagnosis); *B*: the rate of “positive” FKBP51 IHC tests; *B*|*A* (*B* given *A*): the rate of positive tests when the event is present; *A*|*B* (*A* given *B*): is the rate of event occurring when the test result is positive.

## 5. Conclusions

Our study provides evidence for the existence of an inter-relationship between the overexpression of FKBP51 and the biological aggressiveness of OSCCs. Such a finding seems to prove that these molecules may serve as novel predictive biomarkers for the prognosis of these lethal cancers, shedding new light on the very complex molecular events underlying the neoplastic progression of OSCCs.

## Figures and Tables

**Table 1:** Clinicopathological characteristics of the study population (OP: oropharynx; NOP: non-oropharynx; DOD: dead of disease; W&A: well and alive).

HPV Status				
-		**HPV Positive**	**HPV Negative**	**Sub-Total**
		**(% Sub-Total)**	**(% Sub-Total)**	**(% Total)**
		6 (8.3%)	66 (91.7%)	72 (100%)
Gender	male	4 (10%)	36 (90%)	40 (55.6%)
	female	2 (6.3%)	30 (93.8%)	32 (44.4%)
Age	Mean	59.5	64.1	63.8
	Range	44–72	29–89	29–89
Tumor stage	T1	0	10 (100%)	10 (13.9%)
	T2	2 (8%)	23 (92%)	25 (34.7%)
	T3	0	2 (100%)	2 (2.8%)
	T4	0	30 (100%)	30 (41.7%)
	unknown	4 (80%)	1 (20%)	5 (6.9%)
Nodal stage	Nx	-	6 (100%)	6 (8.3%)
	N0	1 (3.6%)	27 (96.4%)	28 (38.9%)
	N1	1 (6.7%)	14 (93.3%)	15 (20.8%)
	N2	-	18 (100%)	18 (25.0%)
	N3	-	1 (100%)	1 (1.4%)
	unknown	4 (100%)	0	4 (5.6%)
Stage	I	-	8 (100%)	8 (11.1%)
	II	1 (6.7%)	14 (93.3%)	15 (20.8%)
	III	1 (16.7%)	5 (83.3%)	6 (8.3%)
	IV	-	38 (100%)	38 (52.8%)
	Unknown	4 (80%)	1 (20%)	5 (6.9%)
Histological tumor differentiation	Poor	-	40 (100%)	40 (55.6%)
	Moderate	-	23 (100%)	23 (31.9%)
	Well	-	3 (100%)	3 (4.2%)
	unknown	6 (100%)	-	6 (8.3%)
Anatomical primary site of tumor	OP	6 (23.1%)	20 (76.9%)	26 (36.1%)
	NOP	0	46 (100%)	46 (63.9%)
Follow-up	DOD	-	32 (100%)	32 (44.4%)
	W&A	6 (18.2%)	27 (81.8%)	33 (45.8%)
	Unknown	-	7 (100%)	7 (9.7%)

**Table 2:** ROC curves analysis: the area under the ROC curves is 0.907 with a 95% CI ranging between 0.806 and 0.966 (*p*-value < 0.0001). At the criterion value >51, the sensitivity and specificity of the test are 88.46 (95% CI 69.8–97.6) and 91.67 (95% CI 77.5–98.2), respectively.

Title	Value
Area under the ROC curve (AUC)	0.907
Standard error	0.0394
95% Confidence interval	0.806–0.966
z Statistic	10.332
Significance level P (Area = 0.5)	<0.0001
Youden index J	0.8013
Associated criterion	>51
Sensitivity	88.46
Specificity	91.67

**Table 3:** ANOVA test revealing a statistically-significant distribution of FKBP51 positivity between p16^INK4a^-negative and -positive groups (*p* = 0.001).

Source of Variation	Sum of Squares	Degree of Freedom (DF)	Mean Square
Between groups (influence factor)	86,791,323	1	86,791,323
Within groups (other fluctuations)	469,380,677	68	6,902,657
Total	556,172,000	69	-
F-ratio	-	-	12,574
Significance level	-	-	*p* = 0.001

## Abbreviations

FKBP51	FK binding protein 51
SCC	Squamous cell carcinoma
HNC	Head and neck cancer
OSCC	Oral squamous cell carcinoma
HPV	Human papilloma virus
IHC	Immunohistochemistry
